# Nemo-like kinase as a negative regulator of nuclear receptor Nurr1 gene transcription in prostate cancer

**DOI:** 10.1186/s12885-016-2291-4

**Published:** 2016-03-31

**Authors:** Jian Wang, Zhi-Hong Yang, Hua Chen, Hua-Hui Li, Li-Yong Chen, Zhu Zhu, Ying Zou, Cong-Cong Ding, Jing Yang, Zhi-Wei He

**Affiliations:** Sino-American Cancer Research Institute, Key Laboratory for Medical Molecular Diagnostics of Guangdong Province, Dongguan Scientific Research Center, Guangdong Medical University, 1 Xincheng Road, Dongguan, 523808 China; Department of Biochemistry, Liaoning Medical University, 40 Songpo Road, Jinzhou, 121001 China; Department of Obstetrics and Gynecology, Longgang District Central Hospital of Shenzhen, 1228 Longgang Road, Shenzhen, 518116 China

**Keywords:** Prostate cancer, NLK, Nurr1, NF-κB, CREB, CBP

## Abstract

**Background:**

Nurr1, a member of the orphan receptor family, plays an important role in several types of cancer. Our previous work demonstrated that increased expression of Nurr1 plays a significant role in the initiation and progression of prostate cancer (PCa), though the mechanisms for regulation of Nurr1 expression remain unknown. In this study, we investigated the hypothesis that Nemo-like kinase (NLK) is a key regulator of Nurr1 expression in PCa.

**Methods:**

Immunohistochemistry and Western blot analysis were used to evaluate levels of NLK and Nurr1 in prostatic tissues and cell lines. The effects of overexpression or knockdown of Nurr1 were evaluated in PCa cells through use of PCR, Western blots and promoter reporter assays. The role of Nurr1 promoter *cis* element was studied by creation of two mutant Nurr1 promoter luciferase constructs, one with a mutated NF-κB binding site and one with a mutated CREB binding site. In addition, three specific inhibitors were used to investigate the roles of these proteins in transcriptional activation of Nurr1, including BAY 11–7082 (NF-κB inhibitor), KG-501 (CREB inhibitor) and ICG-001 (CREB binding protein, CBP, inhibitor). The function of CBP in NLK-mediated regulation of Nurr1 expression was investigated using immunofluorescence, co-immunoprecipitation (Co-IP) and chromatin immunoprecipitation assays (ChIPs).

**Results:**

NLK expression was inversely correlated with Nurr1 expression in prostate cancer tissues and cell lines. Overexpression of NLK suppressed Nurr1 promoter activity, leading to downregulation of Nurr1 expression. In contrast, knockdown of NLK demonstrated opposite results, leading to upregulation of Nurr1. When compared with the wild-type Nurr1 promoter, mutation of NF-κB- and CREB-binding sites of the Nurr1 promoter region significantly reduced the upregulation of Nurr1 induced by knockdown of NLK in LNCaP cells; treatment with inhibitors of CREB, CBP and NF-κB led to similar results. We also found that NLK directly interacts with CBP, that knockdown of NLK significantly increases the recruitment of CBP to both NF-κB- and CREB-binding sites, and that regulation of NLK on Nurr1 expression is abrogated by knockdown of CBP.

**Conclusions:**

Our results suggest that NLK inhibits transcriptional activation of Nurr1 gene by impeding CBP’s role as a co-activator of NF-κB and CREB in prostate cancer.

## Background

In both Europe and the United States, prostate cancer (PCa) is a common malignancy and the leading cause of cancer-associated death among men [1, 2]. Although the incidence of PCa is still lower in Asian than in Western countries, it has been rapidly increasing in recent years due to a more Westernized lifestyle [3]. For patients with localized or regional disease, the prognosis is good; however, median survival in patients with clinically detectable metastasis is only 12–15 months [4–6]. Unfortunately, the exact mechanisms involved in PCa initiation and progression are still unclear, highlighting the need to identify novel biomarkers and clinically-applicable molecular targets for the diagnosis, monitoring and treatment of this disease [7].

The nuclear receptor Nurr1 is a transcription factor belonging to the superfamily of nuclear steroid hormone receptors [8]. It is involved in a wide variety of biological processes, including regulation of proliferation, apoptosis, migration and differentiation in a cell type-specific manner [9–13]. In recent years, the oncogenic functions of Nurr1 have been reported in several types of cancer [11, 14, 15]. In fact, our previous work suggests that upregulation of Nurr1 provides a selective advantage in the initiation and progression of PCa, and that knockdown of Nurr1 inhibits proliferation, migration and invasion of PCa cells and also induces their apoptosis [16]. Taken together, these results suggest that Nurr1 may be a novel marker of PCa, though the mechanisms behind Nurr1 upregulation remain unknown.

Nemo-like kinase (NLK), an evolutionarily conserved serine/threonine kinase, is a member of the mitogen-activated protein kinase (MAPKs) [17, 18]. Recent studies show that NLK expression is altered in various types of human cancer, acting as either an oncogene or tumor suppressor depending upon tumor type [19–21]. In PCa, NLK appears to act as a tumor suppressor, with mRNA expression decreasing concomitant with the development of cancer [22]. NLK may perform this tumor suppressive function in PCa through its suppression of the NF-κB- and CREB-mediated transcription [23–25], and interestingly, the promoter region of the human Nurr1 gene contains both NF-κB- and CREB-binding sites [26]. This data led us to hypothesize that NLK regulates Nurr1 expression in PCa. Here we report for the first time that Nurr1 expression is inversely correlated with NLK in PCa tissues and cell lines, and that transcriptional activation of the Nurr1 gene is repressed by NLK in PCa cells.

## Methods

### Tissue samples

Human PCa specimens were derived from patients undergoing radical prostatectomy, and the benign prostate tissue samples were derived from benign prostatic hyperplasia (BPH) patients. Specimens were formalin-fixed and paraffin-embedded for histopathologic diagnosis and immunohistochemical analysis. Eight paired fresh specimens were frozen in liquid nitrogen immediately following surgical removal and maintained at −80 °C until use (Western blots). Patient clinical features, including age, Gleason score and tumor node metastasis (TNM) staging, are shown in Table 1. This study was approved by the ethics committee of Jinzhou People’s Hospital. Written informed consent for anonymized use of tissue specimens was obtained from all patients.

**Table 1:** Correlation between NLK expression and clinicopathological parameters of PCa patients

No		NLK protein expression (%)		*p*
		High	Low	
Group				
BPH	50	31 (62.0)	19 (38.0)	.003
PCa	118	44 (37.3)	74 (62.7)	
Age (year)				
<70	45	14 (31.1)	31 (68.9)	.276
≥70	73	30 (41.1)	43 (58.9)	
Gleason score				
4–6	42	28 (66.7)	14 (33.3)	<.001
7	20	7 (35.0)	13 (65.0)	
8–10	56	9 (16.1)	47 (83.9)	
CS (TNM)				
T stage				
T_1_-T_2_	56	28 (50.0)	28 (50.0)	.007
T_3_-T_4_	62	16 (25.8)	46 (74.2)	
N stage				
N_0_	90	39 (43.3)	51 (56.7)	.015
N_1_	28	5 (17.9)	23 (82.1)	
M stage				
M_0_	94	39 (41.5)	55 (58.5)	.065
M_1_ (bone)	24	5 (20.8)	19 (79.2)	
Nurr1 expression				
High	84	18 (21.4)	66 (78.6)	<.001
Low	34	26 (76.5)	8 (23.5)	

Statistical analyses were performed by the Pearson *χ* ^2^ test, *p* < 0.05 was considered significant

### Immunohistochemistry

Human prostate tissues were processed for immunostaining with rabbit anti-Nurr1 polyclonal antibody (2 μg/ml, Santa Cruz, CA, USA) and rabbit anti-NLK polyclonal antibody (4 μg/ml, Abcam, Cambridge, MA, USA) as described previously [16]. Stained tissue sections were scored independently by two pathologists blinded to the clinical parameters. The intensity of staining was evaluated subjectively on a scale of 0–3, where 0 = no staining, 1 = weak equivocal staining, 2 = unequivocal moderate staining and 3 = strong staining. The extent of staining, defined as the percentage of positive staining cells in relation to the whole field, was scored on a scale of 0 to 4 as 0 (≤5 %), 1 (6 to 25 %), 2 (26 to 50 %), 3 (51 to 75 %) or 4 (>75 %). Values for staining intensity and positive area were multiplied to give the final score. For statistical analysis, final staining scores of < 4 and ≥ 4 were considered to be low and high expression levels, respectively.

### Cell culture

Three prostate cell lines (LNCaP, PC-3 and BPH-1) were obtained from American Type Culture Collection (Rockville, MD, USA). Cells were cultured in RPMI-1640 media (Invitrogen, Carlsbad, CA, USA) supplemented with 100 IU/mL penicillin G sodium, 100 mg/mL streptomycin sulfate and 10 % (v/v) fetal bovine serum (Hyclone, Logan, UT, USA). Cultures were maintainedat 37 °C in a 5 % CO_2_ environment.

### Small-interfering RNA knockdown

NLK expression in PCa cells was knocked down using NLK-specific shRNA plasmid vectors. The shRNA-NLK (target sequence: 5′-GAATATCCGCTAAGGATGC-3′), shRNA-CBP (target sequence: 5′-AACAGTGGGAACCTTGTTCCA-3′) and shRNA-control plasmids were obtained from GenePharma (Shanghai, China). Cells were grown to 70 % confluency in 6-well plates and transfected with 2 μg specific or nonspecific shRNA plasmid using Lipofectamine LTX Plus (Invitrogen, Carlsbad, USA) according to the manufacturer′s instructions. Cells were harvested 48 h after transfection and used in further experiments.

### Protein expression from plasmid vectors

The human NLK cDNA clone was obtained from Sino Biological Inc (Beijing, China). The NLK coding sequence was amplified by PCR using the following primers: 5′-GAGCGGATAACAATTTCACACAGG-3′ (forward) and 5′-CGCCAGGGTTTTCCCAGTCACGAC-3′ (reverse). The resultant PCR fragment was cloned into the pcDNA3.1 expression vector using the HindIII and XbaI restriction sites. Proper insertion of the clone was confirmed by DNA sequencing, and the plasmid was prepared for transfection using the Genopure Plasmid Maxi Kit (Roche, Basel, Switzerland). Transfection was performed using the Lipofectamine LTX Plus (Invitrogen, Carlsbad, USA) according to the manufacturer’s protocol. Cells were grown to 70 % confluency in 6-well plates and transfected with pcDNA3.1-NLK vector (1 μg) or pcDNA3.1 vector (1 μg). Cells were harvested and used in further experiments 48 h following transfection.

### Western blotting

Western blots were performed in triplicate as previously described [16]. The following antibodies were used for Western blot: rabbit anti-Nurr1 polyclonal antibody (1:500, Santa Cruz, CA, USA), rabbit anti-NLK polyclonal antibody (1:250, Abcam, Cambridge, MA, USA), rabbit anti-NF-κB p65 polyclonal antibody (1:200, Santa Cruz, CA, USA), mouse anti-IκBα monoclonal antibody (1:1000, Abcam, Cambridge, MA, USA), mouse anti-CREB monoclonal antibody (1:500, Santa Cruz, CA, USA), rabbit anti-phospho-CREB polyclonal antibody (Ser133; 1:100, Santa Cruz, CA, USA), mouse anti-β-tubulin monoclonal antibody (1:500, Santa Cruz, CA, USA), rabbit anti-Histone H3 polyclonal antibody (1:250, Santa Cruz, CA, USA), mouse anti-β-actin monoclonal antibody (1:1000, Santa Cruz, CA, USA) and mouse anti-CBP monoclonal antibody (1:500, Abcam, Cambridge, MA, USA). Band density was measured with Image J software and normalized against internal control levels.

### Real-time quantitative RT-PCR

Total RNA was isolated from cultured cells 48 h post-transfection using TRIzol reagent (Invitrogen, Carlsbad, CA, USA) according to the manufacturer’s instructions. Reverse transcription was carried out using the iScript^TM^ cDNA synthesis kit (Bio-Rad Laboratories Inc., Hercules, CA, USA). Fluorescent real-time PCR was carried out in the Bio-Rad iCycler System (Bio-Rad) with a final volume of 10 μl for each reaction. The following primers were used: 5′-TCCAACGAGGGGCTGTGCG-3′ (forward) and 5′-CACTGTGCGCTTAAAGAAGC-3′ (reverse) for human Nurr1; and 5′-GAAGGTGAAGGTCGGAGTC-3′ (forward) and 5′-GAAGATGGTGATGGGATTTC-3′ (reverse) for human GAPDH (internal control). The PCR program was as follows: initial cycle of 95 °C for 3 min, followed by 40 cycles of 95 °C for 15 s, 59.5 °C for 60 s. PCR products were detected using the fluorescent dye SYBR Green I (BioTeke, Beijing, China), and threshold cycle (C_t_) values were generated after every cycle in the run. Fluorescent readings from real-time PCR were quantitatively analyzed by finding the difference between C_t_ values (delta C_t_) of the target and its internal control, with target gene expression determined using the formula 2^-delta Ct^ [25]. This experiment was repeated three times.

### Luciferase reporter gene assay

A fragment of the human Nurr1 promoter region was amplified by PCR using specific primers (forward 5′-AGTTGGTGGGCACAGAGGAGTATC-3′ and reverse 5′-TCACGGAGGGAGGGAGCAG-3′), then cloned into the pGL3-Basic luciferase reporter vector (Promega, Madison, USA) and sequenced to confirm generation of the pGL3-Nurr1-promoter plasmid. A site-directed mutagenesis kit (Stratagene, La Jolla, CA) was used to mutate the core GA sequence of the NF-κB-binding site or the core AC sequence of the CREB-binding site in luciferase constructs. Cells were transfected with wild-type (wt) or mutant (mt) Nurr1 promoter luciferase constructs (1 μg) and with the effector plasmids pcDNA3.1 (1 μg), pcDNA3.1-NLK (1 μg), shRNA-control (2 μg) or shRNA-NLK (2 μg). The TK renilla luciferase plasmid (2 ng) was transfected under the same conditions to enable normalization of transfection efficiencies. Twelve hours after transfecting with WT-Nurr1 promoter constructs, cells were treated with DMSO (control, 1 % DMSO in PBS), BAY 11–7082 (NF-κB inhibitor, 10 μM), KG-501 (CREB inhibitor, 25 μM) or ICG-001 (CBP inhibitor, 25 μM). Firefly and renilla luciferase activities were measured 48 h after transfection in cell extracts using the Dual-Luciferase Reporter Assay System (Promega, Madison, USA). This experiment was repeated three times.

### Immunofluorescence microscopy

LNCaP cells grown on coverslips were washed 3 times with PBS, fixed in 4 % paraformaldehyde for 30 min at room temperature and permeabilized with 0.2 % Triton X-100 solution in PBS for 10 min at room temperature. Next, the samples were incubated in 5 % BSA solution in PBS for 1 h at room temperature to saturate non-specific binding sites. Incubation with the primary antibodies, diluted in 5 % BSA solution in PBS, was performed overnight at 4 °C. Primary antibodies were rabbit anti-NLK polyclonal antibody (1:100, Abcam, Cambridge, MA, USA) and mouse anti-CBP monoclonal antibody (1:200, Abcam, Cambridge, MA, USA). The next day, cells were incubated for 1 h at room temperature in secondary FITC-conjugated anti-rabbit and Cy3-conjugated anti-mouse antibodies (1:300, Sigma-Aldrich, St. Louis, MO, USA). Confocal analysis was performed on a Leica TCS SP5 microscope, with excitation wavelengths set to 488 and 543 nm for dual channel imaging.

### Co-Immunoprecipitation (Co-IP)

Five hundred microgramme of protein lysates from LNCaP cells were incubated with 2 μg of rabbit anti-NLK antibody or mouse anti-CBP antibody overnight at 4 °C on an oscillation shaker. Next, 50 μl of suspensions were generated by mixing samplesina 1:1 ratio with protein A-Sepharose beads and then incubated for 2 h at 4 °C with gentle rotation. To ensure specificity of the detected associations, control IPs with rabbit IgG or mouse IgG in place of the primary antibodies were performed under the same conditions. After extensive washing, precipitates were subjected to Western blotting analyses for detection of potential interacting proteins.

### Chromatin immunoprecipitation assay (ChIP)

Chromatin immunoprecipitations were carried out 48 h after transfection with shRNA-NLK using the MAGnify™ Chromatin Immunoprecipitation System (Invitrogen, Carlsbad, USA) according to the manufacturer’s guidelines. Briefly, LNCaP cells (6 × 10^6^) were fixed with formaldehyde and sonicated. Immunoprecipitation was carried out with 5 μg of mouse anti-CBP antibody (Abcam, Cambridge, MA, USA) or mouse IgG at 4 °C overnight with rotation. The immunoprecipitates were washed and eluted, and purified DNA was obtained. Purified immunoprecipitated DNA and input DNA were used as templates for subsequent real-time PCR. ChIP primer sets were checked for linear amplification and designed to amplify two separate regions of the human Nurr1 promoter: NF-κB element forward (5′-TGGGACAGGAAAAGGGAGTA-3′) and reverse (5′-ACAGATGTCCCATGACACGA-3′), spanning nucleotides −633 to −536; and CREB element forward (5′- AACCACCCAAGCTGGCTAC-3′) and reverse (5′-TGGTATATTTCCGACCTGACG-3′), spanning nucleotides −252 to −163.

### Statistical analysis

Results are expressed as mean ± SD and are representative of at least three separate experiments. The student’s t-test was used to determine statistical differences in the means of two columns. The Pearson *χ*^2^ test and Fisher’s exact test were used to analyze the relationship between the expression of NLK and the patient’s various clinicopathological parameters. The nonparametric Spearman’s rank correlation coefficient was used to determine the statistical dependence between the expression of NLK and Nurr1. A *p* value < 0.05 was regarded as statistically significant. All computations were performed using the SPSS 13.0 software.

## Results

### NLK expression is inversely correlated with Nurr1 expression in PCa

**Fig. 1:**
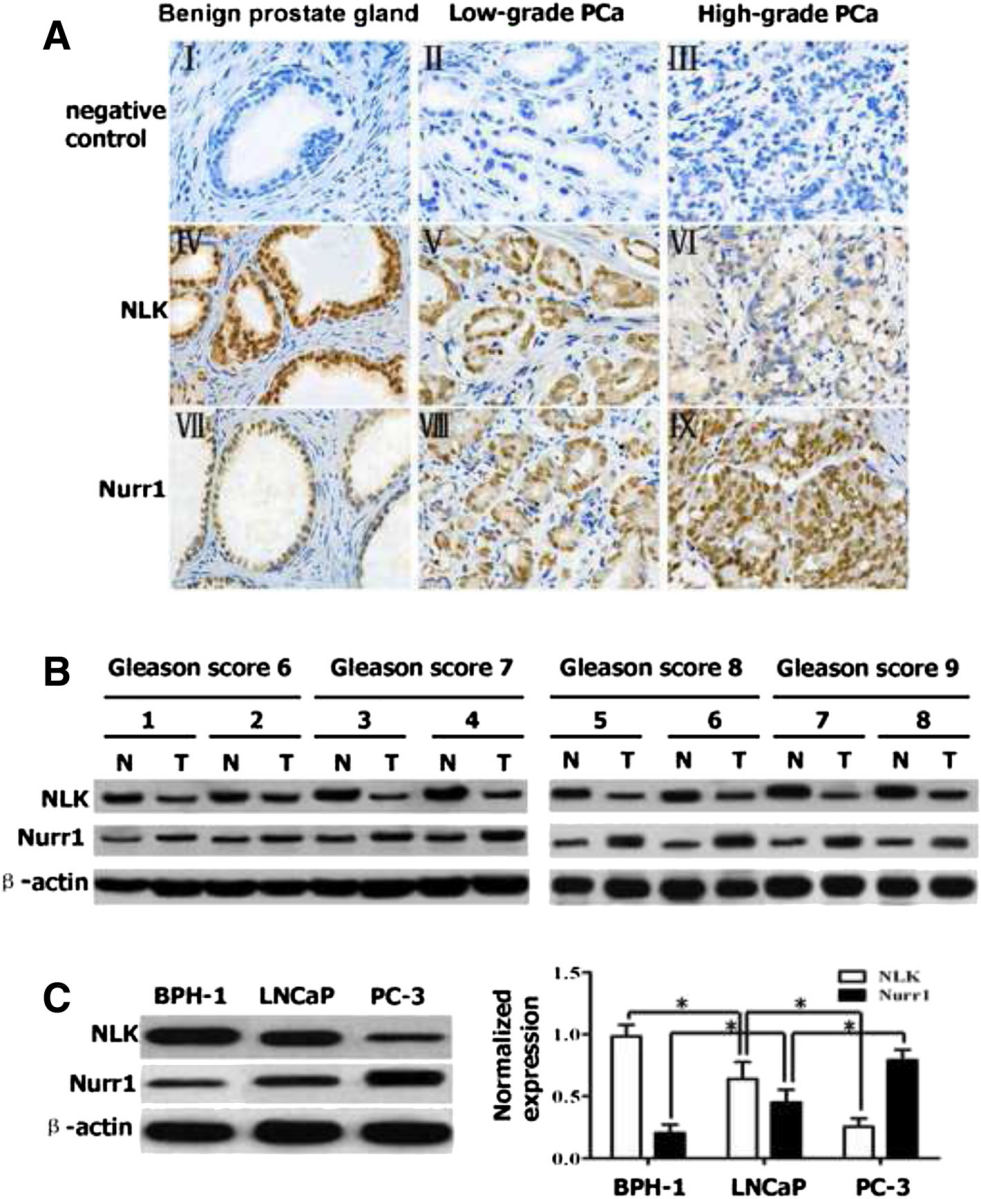
Expression of NLK and Nurr1 in human prostate cancer. **a** (I-IX): Paraffin-embedded tissue sections were stained with antibodies for NLK and Nurr1 and then counterstained with hematoxylin (×400). I-III: Negative controls for benign prostate gland and PCa specimens; IV: High NLK expression in benign prostate gland specimen; V: Medium NLK expression in low-grade PCa specimen; VI: Low NLK expression in high-grade PCa specimen; VII: Low Nurr1 expression in benign prostate gland specimen; VIII: Medium Nurr1 expression in low-grade PCa specimen; IX: High Nurr1 expression in high-grade PCa specimen. **b** Western blotting was performed to study the protein levels of NLK and Nurr1 in eight representative paired samples of PCa tissues (T) and non-tumorous adjacent tissues (N). **c** Western blot analysis of endogenous NLK and Nurr1 in BPH-1, PC-3 and LNCaP cells. Results are plotted as mean ± SD for three independent experiments. *p < 0.05, assessed by Student’s t-test

**Fig. 2:**
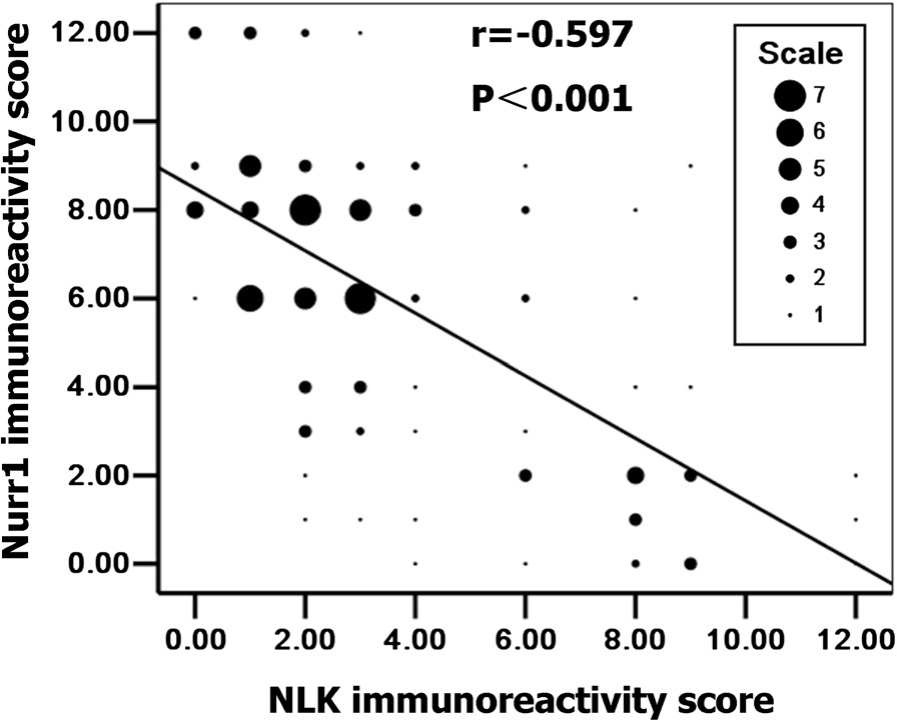
Analysis to study the correlation between NLK and Nurr1 expression in 118 PCa samples by Spearman’s rho method. Spearman’s correlation coefficient by rank test, *r* = −0.597, *p* < 0.001

To explore the clinical significance of NLK in the occurrence and progression of PCa and further characterize the relationship between NLK and Nurr1, we examined the levels of NLK and Nurr1 using immunohistochemical staining in 118 PCa and 50 benign prostate tissue samples. Representative examples of staining are shown in Fig. 1a (I-IX), which show that epithelial cells from benign prostate gland samples have strong nuclear NLK staining (Fig. 1a IV) and weak Nurr1 staining (Fig. 1a VII), and also that low NLK levels (Fig. 1a VI) correlate with high Nurr1 levels (Fig. 1a IX) in the same PCa specimens (high-grade PCa). Correlation analysis demonstrated a significant negative correlation between NLK and Nurr1 expression levels in PCa tissue specimens (Fig. 2). Furthermore, we investigated the abundance of NLK and Nurr1 in eight tumors relative to the adjacent normal tissues (Fig. 1b) by Western blot. The results indicate that compared with the non-tumorous adjacent tissue, NLK expression was dramatically lower and Nurr1 expression much higher in the tumor tissues. To further characterize the relationship between NLK and Nurr1, we investigated their abundance in a normal human prostate epithelial cell line (BPH-1) and two human prostate cancer cell lines (PC-3 and LNCaP) by Western blot analysis. Different expression levels of NLK and Nurr1 were observed in all of the cells (Fig. 1c). As expected, relative abundances of NLK and Nurr1 appeared to be inversely correlated in BPH-1, PC-3 and LNCaP cells. PC-3 cells displayed the lowest abundance of NLK and the highest expression of Nurr1 among the three cell lines.

### Correlation of NLK expression with clinicopathological features in PCa

The frequencies of NLK immunostaining in relation to the different clinicopathological parameters and Nurr1 expression levels are shown in Table 1. For statistical analysis of NLK and Nurr1 expression, the specimens were divided into two groups (high expressers and low expressers), according to staining intensity and the percentages of positive cells. The level of NLK protein was significantly less in the 118 PCa tissue samples compared to the 50 benign prostate tissue samples (*p* < 0.001). In our PCa tissue specimens, the frequency of NLK low expression increased with progressive T classification (*p* = 0.007), N classification (*p* = 0.015) and Gleason score (*p* < 0.001), though no correlation was observed with other prognostic factors such as age and M classification. As expected, NLK expression was negatively correlated with Nurr1 expression in all of the analyzed PCa tissue samples (*p* < 0.001).

### NLK contributes to inhibition of Nurr1 expression in human PCa cells

**Fig. 3:**
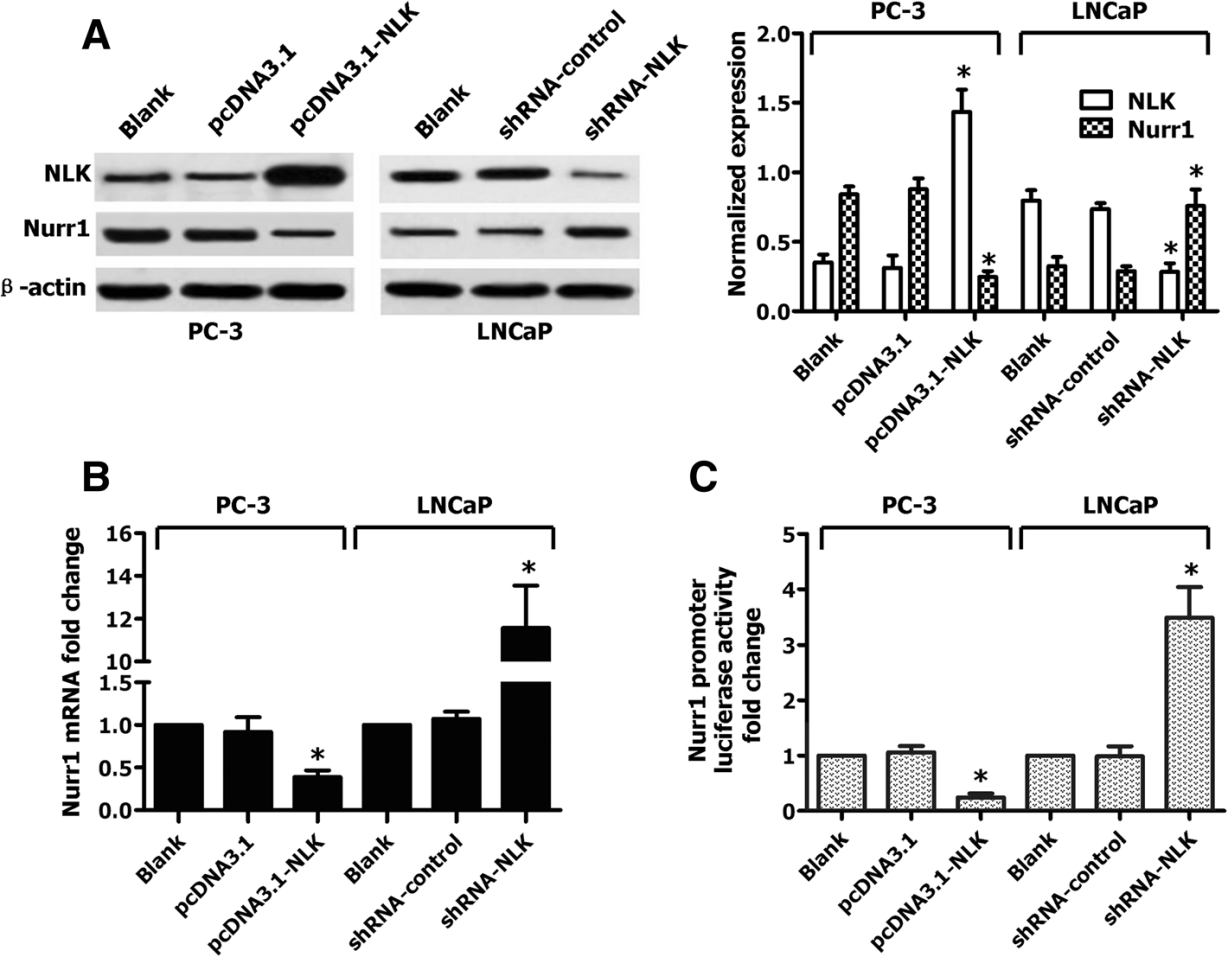
NLK is involved in the suppression of Nurr1 expression in PCa cell lines. PC-3 cells were transfected with NLK-expressing or control vectors, and LNCaP cells were transfected with a shRNA vector targeting NLK or a control nonspecific shRNA vector. **a** 48 h after transfection, Western blot analysis was used to detect protein expression levels of NLK and Nurr1. **b** 48 h after transfection, real-time PCR was performed to detect mRNA levels of Nurr1. **c** Analysis of Nurr1 promoter-luciferase activity was used to determine Nurr1 transcriptional activity. Results are plotted as means ± SD for three independent experiments. *p < 0.05 (compared with control groups), assessed by Student’s t-test

To elucidate the biological importance of NLK in the regulation of Nurr1 gene expression, we used RNA interference to knock down NLK expression in LNCaP cells with a higher level of NLK expression. In contrast, we used transient transfection to upregulate NLK expression in PC-3 cells with lower levels of NLK expression. Levels of Nurr1 protein and mRNA were determined by Western blot analysis and real-time PCR, respectively. As shown in Fig. 3a, b, transfection of the shRNA-NLK vector into LNCaP cells strongly reduced NLK protein levels, whereas shRNA-control vector transfection had no effect on NLK expression. In addition, levels of endogenous Nurr1 mRNA and protein were significantly upregulated by shRNA-NLK transfection. In contrast, NLK protein levels were dramatically increased after transfection of the pcDNA3.1-NLK vector into PC-3 cells, and this upregulation of NLK resulted in a significant downregulation of Nurr1 mRNA and protein expression. These cells were then transfected with the Nurr1 promoter luciferase constructs and either pcDNA3.1-NLK or shRNA-NLK. The results confirm that Nurr1 promoter luciferase activities vary similarly to Nurr1 mRNA and protein levels (Fig. 3c), and strongly suggest that NLK can inhibit Nurr1 promoter activity, thus leading to downregulation of Nurr1 gene expression in human PCa cells.

### Knockdown of NLK in LNCaP cells upregulates Nurr1 promoter activity through both NF-κB- and CREB-binding sites in the Nurr1 promoter region

**Fig. 4:**
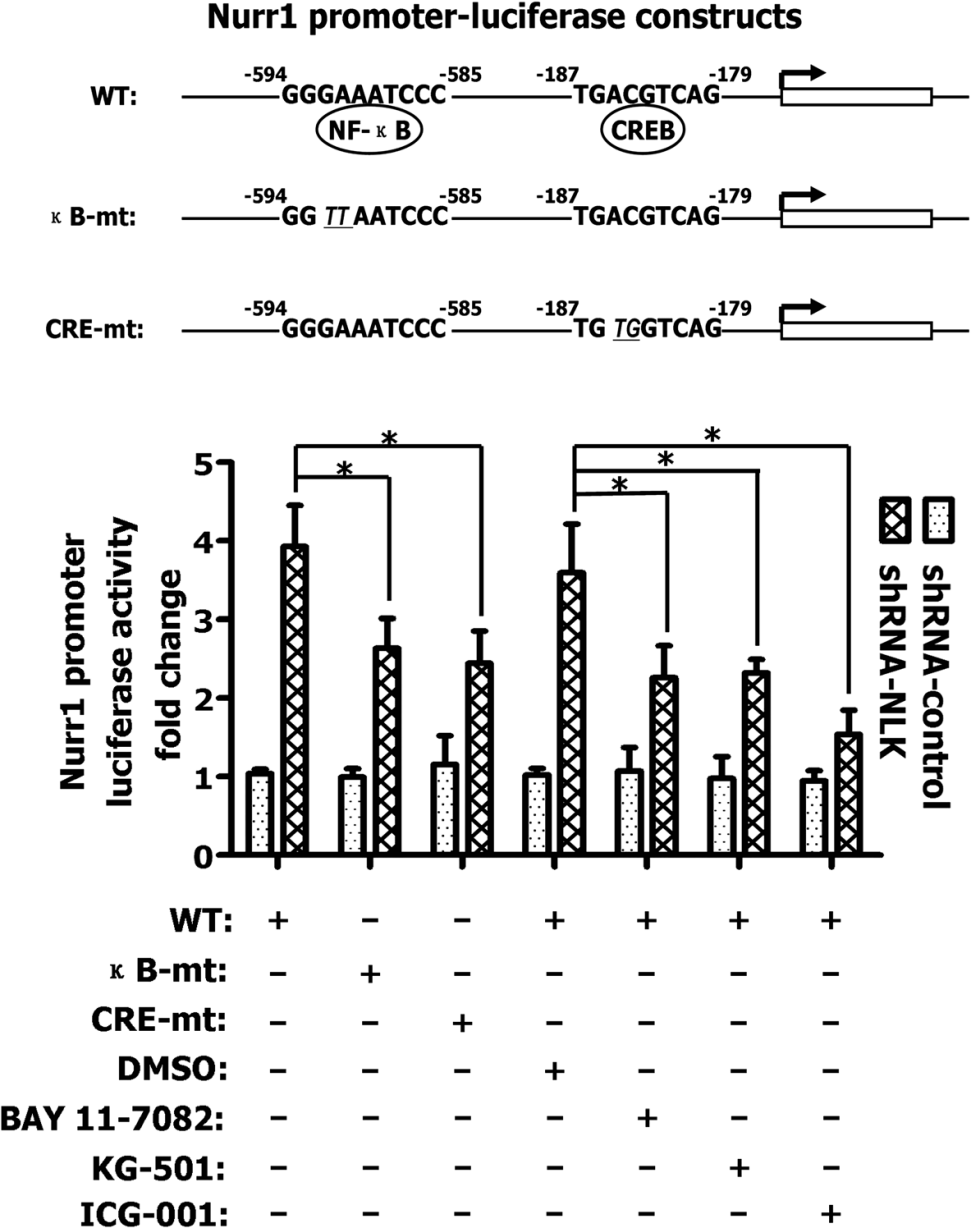
Knockdown of NLK in LNCap cells upregulates Nurr1 promoter activity through both NF-κB- and CREB-binding sites in the Nurr1 promoter region. LNCaP cells were transfected with a shRNA vector targeting NLK and the three Nurr1 promoter luciferase constructs (wild-type or mutant), as indicated. 12 h after transfecting with WT-Nurr1 promoter constructs, cells were treated with DMSO (control), BAY 11–7082 (NF-κB inhibitor), KG-501 (CREB inhibitor) or ICG-001 (CBP inhibitor). Luciferase activity was measured 48 h after transfection. Results are plotted as means ± SD for three independent experiments. *p < 0.05, assessed by Student’s t-test. The illustrated numbers were ratios of data in each group to that in blank control

Analysis of the promoter region of the human Nurr1 gene shows several putative *cis* elements, including the upstream NF-κB-site and the downstream CREB-site [26]. To determine the role of these binding sites in NLK-mediated inhibition of Nurr1 transcription, we created two mutant Nurr1 promoter luciferase constructs (NF-κB-site mutated or CREB-site mutated), as indicated in Fig. 4. Compared to the wild-type Nurr1 promoter construct, mutation of either NF-κB- or CREB-binding sites significantly impaired the upregulation of Nurr1 promoter activity induced by shRNA-NLK transfection into LNCaP cells (Fig. 4). We also used three inhibitors, including BAY 11–7082 (NF-κB inhibitor), KG-501 (CREB inhibitor) and ICG-001 (CREB binding protein, CBP, inhibitor). Figure 4 shows the upregulation of Nurr1 promoter luciferase activity induced by shRNA-NLK under control conditions and following treatment with BAY 11–7082, KG-501 or ICG-001. All three inhibitors markedly inhibited Nurr1 promoter activation induced by shRNA-NLK, indicating that NF-κB- and CREB-mediated transcriptional activation are required for upregulation of Nurr1 gene expression by shRNA-NLK.

### CBP is a potential direct target of NLK in suppression of NF-κB- and CREB-mediated transcription

**Fig. 5:**
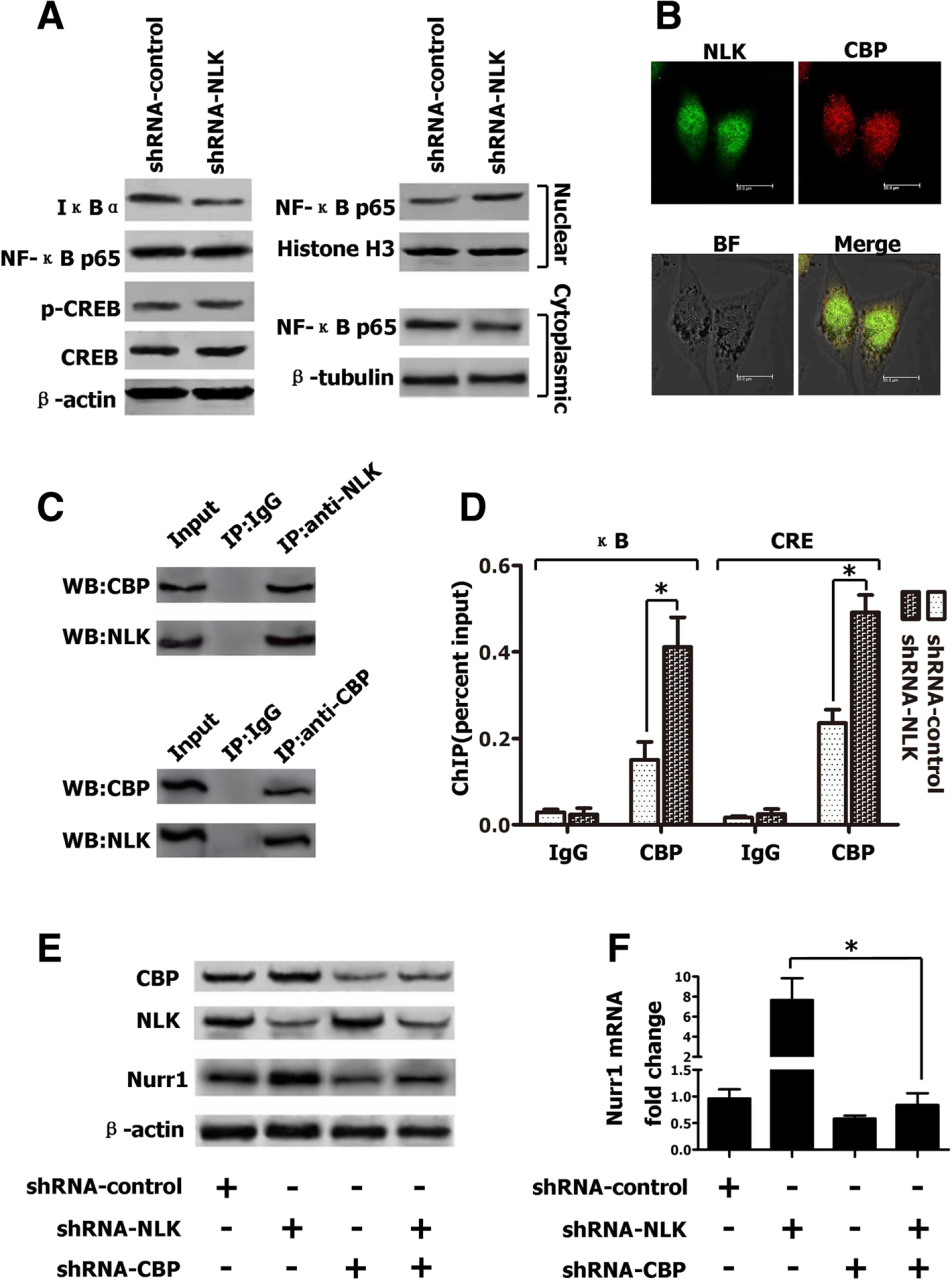
CBP is a potential direct target of NLK in suppression of the NF-κB- and CREB-mediated transcription in LNCaP cells. **a** 48 h after transfection, Western blot analysis was used to detect protein levels of p65, IκBα, CREB and phosphorylated CREB (Ser133). For analysis of the subcellular distribution of p65, LNCaP cells were fractionated into cytoplasmic and nuclear fractions. **b** NLK and CBP are co-localized in the LNCaP cells. Cells were subjected to immunofluorescence with anti-NLK antibody followed by FITC-conjugated antibody (green) and anti-CBP antibody followed by Cy3-conjugated antibody (red). **c** Interaction of NLK with CBP in LNCaP whole cell extract. LNCaP cells were lysed and used for Co-IP assays with anti-NLK, anti-CBP or normal IgG antibody. **d** ChIP analysis of CBP occupancy of the Nurr1 promoter in LNCaP cells transfected with shRNA-control or shRNA-NLK. **e** 48 h after transfection, Western blot analysis was used to detect protein expression levels of CBP, NLK and Nurr1. f: 48 h after transfection, real-time PCR was performed to detect mRNA levels of Nurr1. Data are means ± SD for three independent experiments. *p < 0.05, assessed by Student’s t-test

Having demonstrated that NLK inhibits NF-κB- and CREB-mediated transcriptional activation, we sought to determine changes in the protein levels of p65, IκBα, CREB and phospho-CREB in the presence of shRNA-NLK or shRNA-control. As shown in Fig. 5a, protein levels of p65, CREB and phospho-CREB did not change, whereas IκBα levels decreased. We next performed Western blotting to determine the contents of p65 included in cytoplasmic and nuclear extracts collected from shRNA-NLK- and control-transfected LNCaP cells, which showed that knockdown of NLK increased the level of p65 in nuclear extracts (Fig. 5a). Since ICG-001 (CBP inhibitor) can inhibit Nurr1 promoter activation induced by shRNA-NLK (Fig. 4) and CBP is a nuclear transcriptional co-activator of NF-κB and CREB, we suspected that CBP was a target of NLK in LNCaP cells. To test this hypothesis, dual-color fluorescence and co-immunoprecipitation experiments were used to investigate whether interaction occurs between the two proteins. Immunofluorescent microscopy (Fig. 5b) demonstrated co-localization (yellow) of NLK and CBP in LNCaP cells. Their interaction was further confirmed through immunoprecipitation analysis, which detected the CBP protein in anti-NLK immunoprecipitates and the NLK protein in anti-CBP immunoprecipitates from whole cell lysates (Fig. 5c). To determine if CBP is critical for NF-κB- and CREB-mediated transcriptional activation of Nurr1, we used ChIP to examine the endogenous occupancy of CBP at the Nurr1 promoter in the presence and absence of shRNA-NLK. As shown in Fig. 5d, compared with controls, knockdown of NLK markedly increases the recruitment of CBP on both the NF-κB- and CREB-binding regions. Finally, we employed siRNA approach to knock down CBP expression and examined if CBP was involved in regulation of NLK on Nurr1 expression. As shown in Fig. 5e, f, the regulation of NLK on Nurr1 expression was abrogated by knockdown of CBP in LNCaP cells. Taken together, these results support the notion that NLK directly interacts with CBP to regulate transcriptional activation of Nurr1 in LNCaP cells.

## Discussion

Our previous work revealed that expression of Nurr1 was higher in PCa tissues than in benign prostate tissues, and high levels were positively correlated with ascending TNM classification and Gleason scores for prostate cancer patients [16]. To date no reports have been published on the relationship between NLK and Nurr1. In the present study, we sought to explore this relationship through examination of the expression levels of NLK and Nurr1 in the same paraffin-embedded tissue samples. In contrast to expression patterns of Nurr1, levels of NLK protein were significantly reduced in PCa samples compared to benign prostate tissue samples, with low NLK expression correlated with progression of T and N classification and Gleason scores. Previous studies have not found significant differences in NLK protein expression between normal prostatic tissues and primary PCa when evaluated by immunohistochemical staining [22], though this may be due to small sample size. We also observed an inverse correlation between the expression of NLK and Nurr1 by investigating the expression of NLK and Nurr1 in three human prostate cell lines and eight tumors relative to their adjacent normal fresh tissues. These data provide direct evidence for an inverse relationship between NLK and Nurr1 expression in PCa. We next examined the role of NLK in the regulation of Nurr1 expression by reducing NLK expression through RNA interference or upregulating NLK expression by transfection of NLK-expressing plasmid vectors into PCa cells. Consistent with our previous results, up- or down-regulation of NLK expression dramatically influenced protein and mRNA levels, and the promoter activity of Nurr1. This suggests that NLK may inhibit Nurr1 promoter activity, thus leading to downregulation of Nurr1 expression in human PCa cells.

DNA sequence analysis of the human Nurr1 promoter revealed several potential regulatory regions, including binding sites for NF-κB, CREB and Sp1 [26]. Previous studies have demonstrated the involvement of CREB in the transcription of Nurr1 in Hela cells, lung cancer cells and HUVEC cells [27–29]. Other studies have also shown that enhanced NF-κB binding of the Nurr1 promoter is a important mechanism in the regulation of Nurr1 transcription [30, 31]. To elucidate the role of these binding sites in Nurr1 transcription, we mutated NF-κB- and CREB-binding sites of Nurr1 promoter luciferase constructs and used three specific inhibitors of these proteins. Our results suggested that upregulation of Nurr1 gene expression induced by shRNA-NLK depends on NF-κB- and CREB-binding sites for transcriptional activation in LNCaP cells.

NF-κB is a nuclear transcription factor that binds to NF-κB response elements within target genes [32, 33]. Under resting conditions, NF-κB is maintained as a latent complex in the cytoplasm by the inhibitory IκB proteins [34]. In the canonical activation pathway, IκBα is phosphorylated by the IκB kinase (IKK) complex. Phosphorylation of IκBα on serines 32 and 36 triggers its ubiquitination and proteasome-dependent degradation, thus permitting the translocation of bound NF-κB dimers to the nucleus and gene expression activation [35]. In the present study, we found that levels of IκBα were reduced, and that the nuclear:cytoplasmic ratio of p65 protein was increased following induction by shRNA-NLK. A previous study showed that NLK competes with TAK1 to bind IKKβ, leading to inhibition of activation by the IKKβ phosphorylation and IκBα degradation [36]. Presumably, a similar mechanism may be responsible for nuclear translocation of p65 by NLK knockdown in LNCaP cells. However, we found this nuclear translocation is not strong (Fig. 5a). Moreover, NF-κB-mediated transcriptional activation requires the help of its coactivators in the nucleus [37], so the NLK in nucleus may be more important than those in cytoplasm in the regulation of Nurr1 gene transcription.

CREB was one of the first transcription factors purified by DNA affinity chromatography using the cyclic AMP binding element (CRE) of the somatostatin gene [38]. Activation of CREB is mediated by phosphorylation at its serine 133 residue, which promotes the association of CREB with CBP/p300, leading to transcriptional activation of its target genes through histone modification and recruitment of an active transcription complex [39, 40]. Our results indicate that knockdown of NLK does not affect the levels of expression and phosphorylation of the CREB protein, suggesting that CREB itself is not a direct target of NLK that results in its transcriptional suppression.

We found that the CBP inhibitor ICG-001 markedly inhibited NLK knockdown- induced Nurr1 promoter activation, suggesting that CBP may participate in NLK-mediated repression of Nurr1 promoter activity. CBP and its homologue p300 possess intrinsic histone acetyltransferase (HAT) activity, and are also transcriptional co-activators of various sequence-specific transcription factors such as NF-κB, AP-1, Smad and p53 [41–44]. CBP/p300 can enhance transcriptional activity either through their protein acetyltransferase activity or by acting as scaffold proteins to recruit other coregulators or components of the basal transcription machinery [45–47]. CBP/p300 functionally interact with a region of the NF-κB p65 subunit containing the transcriptional activation domain and potentiates p65-activated transcription [48]. Thus, recruitment of CBP/p300 to transcription factors is required for NF-κB- and CREB-mediated transcriptional activation. A previous study indicated that NLK may regulate protein-protein interactions mediated by phosphorylation of the C-terminal region of CBP and suppress its function. NLK carries out this function to repress CBP through its kinase-dependent activity [23]. Our study demonstrated that NLK and CBP physically interact, suggesting that NLK may inhibit NF-κB- and CREB-mediated transcriptional activation through downregulation of NF-κB-CBP/p300 and CREB-CBP/p300 complexes. ChIP analysis further demonstrated that NLK knockdown promotes recruitment of CBP to both NF-κB- and CREB-binding regions of the Nurr1 promoter. Taken together, these evidences suggest that CBP is likely to be an important direct target of NLK in the regulation of Nurr1 expression in prostate cancer.

Although the activation of AR participated in the activation of NF-κB signaling pathway [49], it is not the only cause for the activation of this pathway [50, 51]. In addition, no androgen-treated cells were used in the present study. Hence, we speculate that although we used two cell lines with different characteristics of androgen independency, the results will not be substantially affected. The aim of this study was to elucidate the mechanisms underlying the regulation of transcription of Nurr1 by NLK. The association between androgen and Nurr1 could not be determined and will be investigated in our future studies. We speculate that regulation of expression of Nurr1 in prostate cancer could be androgen-independent or partially-dependent.

## Conclusions

**Fig. 6:**
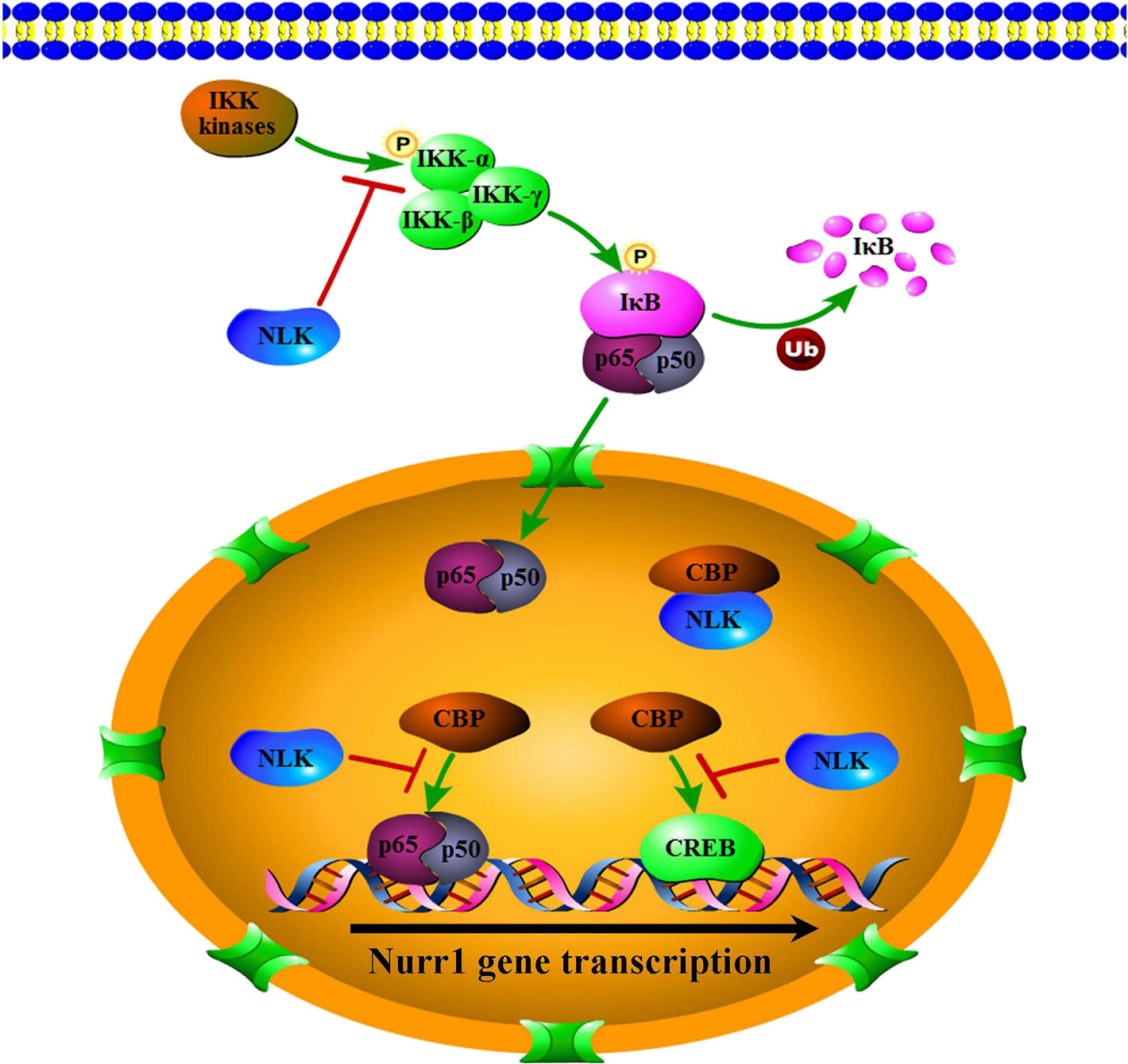
Schematic representation of the proposed signaling pathways for NLK-inhibited Nurr1 expression in PCa cells

NLK is a transcriptional repressor of Nurr1 expression in human PCa. Reduced expression of NLK may attenuate inhibition of CBP function as co-activator of NF-κB and CREB, thus promoting transcriptional activation of Nurr1 gene (Fig. 6). Further studies are needed to delineate the effect and precise mechanisms of the NLK/Nurr1 signaling pathway in PCa development and progression, and also to demonstrate the value of this pathway as a therapeutic target.

## Abbreviations

BAY 11–7082: NF-κB inhibitor
CBP: CREB binding protein
ChIP: chromatin immunoprecipitation assay
Co-IP: co-immunoprecipitation
CREB: cAMP-response element binding protein
HAT: histone acetyltransferase
ICG-001: CBP inhibitor
IKK: IκB kinase
IκB: inhibitor of NF-κB
KG-501: CREB inhibitor
MAPKs: mitogen-activated protein kinase
NF-κB: nuclear factor-kappaB
NLK: Nemo-like kinase
PCa: prostate cancer
TAK1: TGF-β-activated kinase 1
